# Complexity measure based on sensitivity analysis applied to an intensive care unit system

**DOI:** 10.1038/s41598-023-40149-x

**Published:** 2023-09-05

**Authors:** Joao R. B. Paiva, Viviane M. G. Pacheco, Poliana S. Barbosa, Fabiana R. Almeida, Gabriel A. Wainer, Flavio A. Gomes, Antonio P. Coimbra, Wesley P. Calixto

**Affiliations:** 1 School of Electrical, Mechanical and Computer Engineering (EMC), Federal University of Goias (UFG), Goiania, GO 74605-010 Brazil; 2 Studies and Researches in Science and Technology Group (GCITE), Federal Institute of Goias (IFG), Goiania, GO 74130-012 Brazil; 3 Visualization and Simulation Centre (VSIM), Carleton University (CU), Ottawa, ON K1S 5B6 Canada; 4 Systems and Robotics Institute (ISR), Coimbra University (UC), 3030-790 Coimbra, DC Portugal

**Keywords:** Biomedical engineering, Health care, Engineering

## Abstract

This work proposes a system complexity metric and its application to Intensive Care Unit (ICU) system. The methodology for applying said complexity metric comprises: (i) parameters sensitivity indices calculation, (ii) mapping connections dynamics between system components, and (iii) system’s complexity calculation. After simulating the ICU computer model and using the proposed methodology, we obtained results regarding: number of admissions, number of patients in the queue, length of stay, beds in use, ICU performance, and system complexity values (in regular or overloaded operation). As the number of patients in the queue increased, the ICU system complexity also increased, indicating a need for policies to promote system robustness.

## Introduction

In critical care, we usually need to connect diverse interdependent entities to build a cohesive system capable to provide health recovery to patients. Those characteristics make the Intensive Care Unit (ICU) a complex system whose behavior is determined by rules that may change according to different needs^1–3^ (for instance, the different medical protocols). From admission to minimum operating conditions, multidisciplinary teams work cooperatively and effectively to achieve goals related to critical or potentially seriously sick patients recovery. In addition to human resources, critical care units need various devices, equipment and medicine, and this represents a significant cost for hospital management. Measuring ICU complexity can be useful to understand the health system dynamics in the intensive care context, and the impact of small changes in some variables.

To address critical care mechanisms complexity, we need to see health care reality as a set of heterogeneous components that act as a whole. According to Morin^4^, complexity is a tissue of heterogeneous constituents inseparably associated or the tissue of events, actions, interactions, feedback, determinations, and accidents that make up our phenomenal world.

Nature itself is characterized by complex organization patterns that combine regularity and randomness in its structure and behavior^5,6^. Recurring patterns are often found in nature’s ever-changing configurations, and even a limited number of rules or laws may produce complex structures, e.g. DNA consists of strings of the same four nucleotides, yet no two individuals are exactly alike. This characteristic is known as perpetual novelty and is present in most complex systems^3^. For some decades, several studies have been addressing complexity as the quality of being complex or as a scientific field with several branches^3,7–10^. These studies identify that complex systems may have some of these characteristics: non-linearity, emergence, self-organization, diversity, interdependence, evolution, and perpetual novelty, among others.

In intensive care context, we focus on two aspects: diversity and interdependence. From materials to people, we may observe the diversity of intensive care services in terms of devices, equipment, drugs, procedures, and professionals (physicians, residents, nurses, physiotherapists, and technicians). According to Page^1^, diversity may enhance the robustness of complex systems maintaining their functionality, in addition to driving innovation and productivity. However, if one part strongly coupled with others is affected, the whole system may be compromised^11,12^.

By observing these aspects in the ICU environment, we may analyze: (i) the diversity based on resources needed for intensive care and (ii) the interdependence based on adverse events cases. In addition to the impact on patients physical integrity, adverse events result in increased healthcare costs due to longer hospital stays; they affect health professionals psychologically and undermine confidence in medical staff. These implications have led to studies on safety culture that ensure early identification and prevention of major groups of adverse events^13,14^.

Forster et al.^15^ investigated whether adverse events are associated with length of stay at ICU and mortality. Adverse events were observed in $19\%$ of ICU patients and refer to (i) procedural complication, (ii) nosocomial infection, (iii) adverse drug event, (iv) surgical complication, (v) therapeutic error, (vi) system error, and (vii) diagnostic error. The number of preventable events was 6/18, 8/13, 2/12, 0/6, 5/5, 1/1 and 1/1, respectively. As research findings, adverse events were independently associated with an average increase in hospital length of stay of 31 days, however a significant statistical association between adverse events and mortality was not found.

As the length of stay at the ICU increased, the number of available beds decreased, which affects new patients admission. Using queuing theory and sensitivity analysis, McManus et al.^16^ assessed the impact of bed unavailability on ICU performance in a pediatric hospital during a 2-year period. Based on the admission, discharge and turn-away data, the authors observed that the rejection rate increased exponentially as ICU utilization exceeded $80\%$. This behavior was confirmed from results given by the queuing model, which also allowed to predict system performance from changes in unit size.

The ICU system overload makes the critical care environment more susceptible to errors or negligence, requiring greater staff attention and cooperation. Based on a game-theoretic experiment, Guazzini et al.^17^ verified that humans accurately estimate the benefits of collaboration when facing hard problems. Thus the staff’s behavioral responses in the ICU environment may change according to the situation complexity.

Although there are studies on the intensive care complexity^2,18,19^, none of them applies complexity metrics and sensitivity analysis at the same time. Considering the restricted number of resources in intensive care, stay extension at the ICU due to adverse events, financial costs for the health system, psychological and physical problems associated with adverse events or bed unavailability, this paper presents a quantitative study on ICU complexity and applies sensitivity analysis for defining the relevance of the connections between elements. We propose a complexity metric that considers both internal and external factors, measuring the impact generated on outputs due to parameters variation, such as resources and patient arrival rate, to use sensitivity indices as connection weights.

In addition to the complexity metric, this work presents an ICU performance metric and the relationship between complexity, performance, and workload. The proposed complexity measure encompasses different aspects of the system: (i) arrangement, based on connections, (ii) configuration, obtained by adjusting the parameters, (iii) performance, used as system output in the calculation of sensitivity indices and, (iv) workload, checked by the number of connections.

## Theoretical background

### Systems, models and simulation

Systems are composed of units that interact with each other through connections and with the environment through boundary components. These units or system components working together achieve results that could not be obtained by individual components^20–22^. Systems have the quality of encompassing subsystems and being encompassed by larger systems at the same time^12,23^. Thus, systems are differentiated by their properties, which are: (i) totality – the internal cohesion among elements and boundaries components, (ii) composition – the elements and their interactions, (iii) internal organization – the way its structure performs functions, and (iv) external organization – the interaction rules with the environment^12,23,24^.

System modeling is the process of representing the system through behavior rules and interaction with the environment. Several studies and analyses can be performed using the model^25^. During the modeling process, it is necessary to define which systems details are necessary for the study, without addressing irrelevant particularities. The definition of scope and modeling technique depends on the modeler’s experience and research emphasis^26^.

One of the techniques used to model real systems is Discrete Event System (DES) modeling. This technique refers to the class of systems that depend on events occurrence to evolve. The events are the result of intentional actions or triggered upon verification of a certain condition. These actions promote system state changes at random time intervals^27^. Both the events and states that describe discrete event systems can be mapped into finite discrete sets in the modeling process^26^. Using the model, input parameters (historical, real, or hypothetical) can be manipulated during experiments to observe the outputs^27^. These experiments can be performed using simulation, which aims to reproduce the modeled system dynamics behavior, allowing us to understand its functioning^28^.

### System’s complexity metrics

Systems complexity comprises the system as an interacting whole (interaction between its parts and the whole with the environment). From a behavioral perspective, complexity is a subjective matter, since the system’s behaviors are described by those who observe the system^29,30^. Complex systems have the following characteristics: (i) nonlinearity – the superposition property absence, since only linear relations are represented by partial processes that can be superimposed to obtain the total process^20^, (ii) emergence – the collective behavior of the system elements, which can appear in a small part of the system or in the system as a whole^31^, (iii) self-organization – the ability to establish its own rules and generate new behavior patterns without external intervention^1,32,33^, (iv) diversity – heterogeneity of its member agents, allowing for cooperative relations^1^, (v) interdependence – the level of influence existing between its parts and the whole^11^, and (vi) evolution – the ability to develop trajectory in space and time by recombining structures that compose it^3^.

The literature presents several complexity metrics. Deacon and Koutroufinis^5^ and Gell-Mann^34^ propose that complexity consists of the balance between order and disorder, regularity, and randomness. Considering the system under study, Lloyd^35^ lists forty system complexity metrics in three groups: (i) description difficulty, (ii) creation difficulty, and (iii) degree of organization. Complexity metrics can be based on entropy^36^, degree of hierarchy^10^, algorithmic information content^37,38^, computational capacity^39^, thermodynamic depth^40^, statistics^41^, fractal dimension^42^, logical depth^43^, dynamic depth^5^, amount of information^31^, size^8^, and connections^44^.

### Sensitivity analysis

**Figure 1:**
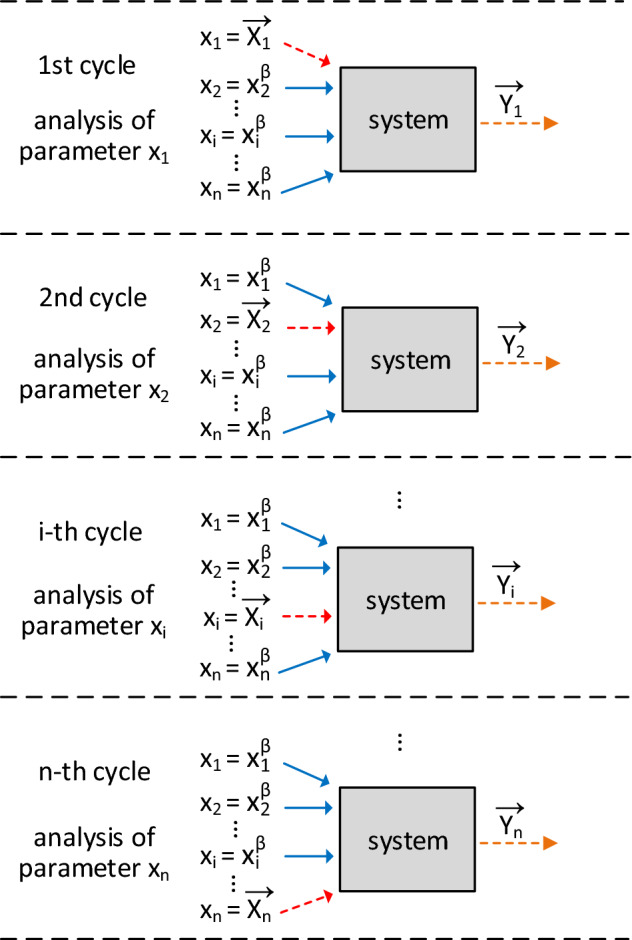
Sensitivity analysis cycles using the one-at-a-time method.

Sensitivity analysis allows measuring the impact generated on system outputs due to variations in input variables^45–47^. Figure 1, adapted from Paiva^48^, illustrates the sensitivity analysis process for a system with *n* input parameters given by $x_{1}, x_{2}, \cdots , x_{i}, \cdots x_{n}$.

Using local sensitivity analysis, we first investigate the parameters reference values that drive the system to its best performance. Thus, we define these reference values set, called the base case $\overrightarrow{\beta }$, given by:

1 $$\begin{aligned} \overrightarrow{\beta } = [x_{1}^{\beta }, x_{2}^{\beta }, \cdots , x_{i}^{\beta }, \cdots , x_{n}^{\beta }], \end{aligned}$$

where $x_{i}^{\beta }$ is the reference value for $x_{i}$ parameter. After defining the parameter base value, we must choose a set of values for each parameter in the range between – 100 and 100% from the base value. In Fig. 1, we can see that the analysis cycle is performed for each system parameter. While modifying one parameter (red arrow), others are kept constant (blue arrows) in their reference values. Thus, we can check the system’s sensitivity to each $x_{i}$ parameter variations. This analysis comprises scenarios definition, that is, different values of the parameter under analysis in the variation range combined with the reference values of other parameters. These scenarios are simulated (or applied to the real system) and the obtained outputs are stored in $\overrightarrow{Y_{i}}$.

**Figure 2:**
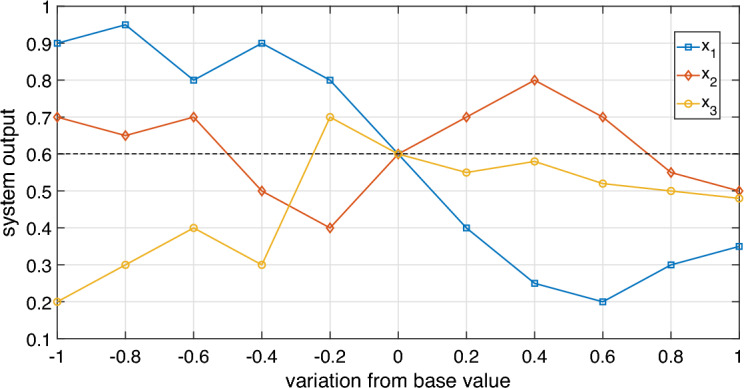
Hypothetical spider-diagram of the system sensitivity analysis with three input parameters.

Eschenbach and McKeague^49^ propose a visual method using a graph called spider diagram. Figure 2, adapted from Pannell^50^, illustrates a spider diagram considering system sensitivity analysis with three input parameters: $x_{1}$, $x_{2}$ and $x_{3}$. Figure 2 shows the output values in the ordinate axis, the input parameters variation from the base value in the abscissa axis, and the ordered pairs corresponding to analyzed scenarios using markers.

In the spider diagram illustrated in Fig. 2, all parameters can be modified in the range of $-100$ to $100\%$ from the base value given by $\overrightarrow{\beta }$. However, in practical situations, some parameters may have physical limitations that restrict this variation range. The more sensitive the parameter (considering the difference between obtained output value and the output value corresponding to $\overrightarrow{\beta }$ scenario), the greater its impact on the system output. Based on this statement, Gomes^51^ proposes the area method to obtain the sensitivity indices of input parameters. The spider diagram analysis interval is defined by the analyst, considering possible parameter restrictions.

The area method consists of the relationship between areas defined by the parameter curves. The axis parallel to the abscissa axis in the spider diagram is called the base axis. Thus, the areas between each parameter curve and the base axis correspond to the parameter contribution in relation to the total area. The parameter’s sensitivity index is given by:

2 $$\begin{aligned} S_{x_i}^{a} = \dfrac{A_{x_i}}{\displaystyle \sum _{i=1}^{n} A_{x_i}}, \end{aligned}$$

where $S_{x_i}^{a}$ is the sensitivity index of $x_{i}$ parameter, $A_{x_i}$ is the area formed by the variation curve of $x_{i}$ parameter and the base axis, *n* is the number of input parameters^51^.

## Methodology

### Proposed complexity metric

**Figure 3:**
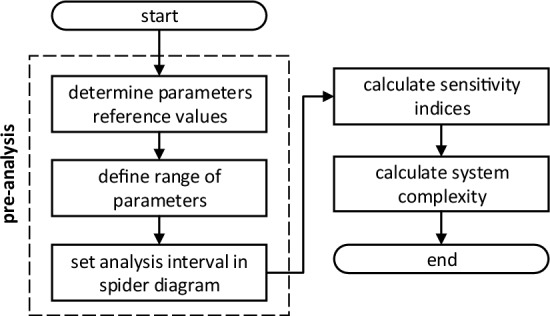
Proposed methodology action flow.

The proposed complexity metric uses sensitivity indices to calculate complexity. These indices are used as connection weights of the system under study. Figure 3 illustrates the steps to apply the proposed metric. Based on the system model, we carry out the pre-analysis, when we define: (i) base scenario or base case, (ii) parameters variation range, and (iii) analysis interval in the spider diagram.

Using the area method, we can calculate the system parameters sensitivity indices, according to expression (2). The output variable can be defined as the system performance. Considering modifying the reference values using the one-at-a-time method, we can get performance values corresponding to different scenarios. Thus, the parameters can be related to the different system connections through sensitivity indices. For example, the connection between components *A* and *B* can be related to parameters $x_2$, $x_3$, and $x_5$. Therefore, the relevance of this connection $\gamma _{c}$ would be equal to the sum of these parameters sensitivity indices ($S_{x_2}^{a}+S_{x_3}^{a}+S_{x_5}^{a }$). Generically, the connection relevance $\gamma _{c_i}$ is obtained by the sum of parameters sensitivity indices $S^a_{x_j}$ directly related to the connection $c_i$, expressed by:

3 $$\begin{aligned} \begin{array}{lll} \gamma _{c_{i}} = \displaystyle \sum _{j=1}^{n}\,S^a_{x_j} &{} \textit{if} &{} x_j\,\textit{is directly related to}\,c_{i}\\ \end{array}. \end{aligned}$$

The use of sensitivity analysis in the metric of system complexity was proposed by Gomes^51^, in which the connection relevance $\gamma _{c}$ corresponds to the connection weight in the system. This metric bases on the entropy conception to quantify the influence of the uncertainty inside the system added to the influence of the uncertainty generated by external elements, given by sensitivity indices. Thus, the system complexity is calculated by:

4 $$\begin{aligned} \psi (c,\gamma ) = \sum _{i=1}^{\rho }\,\left[ \gamma _{c_i}-P(c_i)\cdot \,log_{2}\,P(c_i)\right], \end{aligned}$$

where $\psi (c,\gamma )$ is the system complexity based on weighted connections, $\rho$ is the number of active connections at each instant, $P(c_i)$ is the connection probability $c_i$ to occur, and $\gamma _{c_i}$ is the connection relevance. The connection occurrence probability $P(c_i)$ can be theoretical or experimental, according to the system analysis. The number of active connections $\rho$ is given during system operation.

### Intensive care unit model

**Figure 4:**
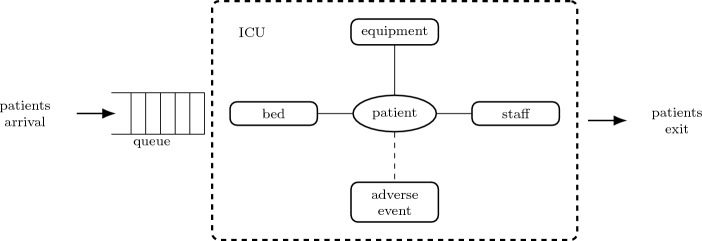
Connection-based ICU model.

The intensive care unit proposed model is defined based on the ICU patient flow. In summary, the model consists of the patients arrival, resource demand, patients permanence in the ICU, adverse events occurrence, and patients exit after being discharged or dying. These relationships can be modeled as connections between system elements, as illustrated in Fig. 4.

**Figure 5:**
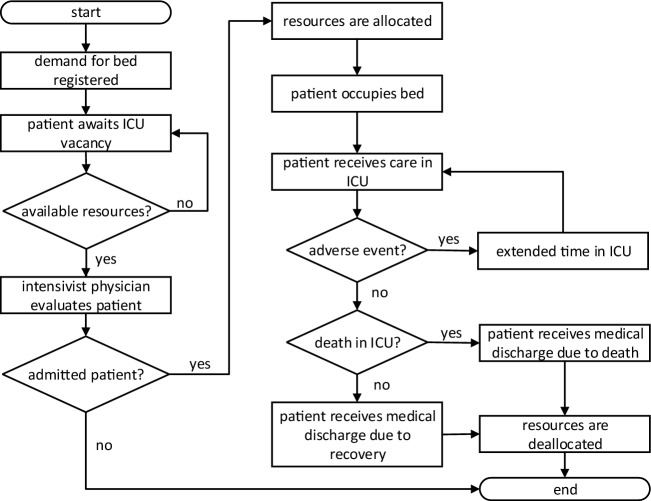
ICU patient flow.

The ICU patient flow starts by requesting an ICU bed. The patient waits in the queue until resources are available and the intensivist physician confirms his/her admission. If the patient is admitted, resources are allocated, and the patient occupies an ICU bed and receives care from staff. If the patient is not admitted, the bed request is canceled. In the case of hospitalization, the length of stay in the ICU is increased whenever an adverse event occurs. During his/her stay in ICU, the patient can die. When the patient is discharged or dies, the resources are deallocated and the patient flow ends, as shown in Fig. 5.

Considering the ICU patient flow shown in Fig. 5, the system dynamics are defined according to probability distributions. The system can be expressed in terms of states and events to perform system simulation of discrete events. The set of discrete states of patients in the intensive care unit is: (i) in queue waiting for admission, (ii) in consultation with an intensivist physician, (iii) refused admission, (iv) hospitalized, (v) recovered, and (vi) deceased. The set of events is: (i) patient arrival (registration of vacancy demand), (ii) evaluation of patient’s clinical status, (iii) refusal of admission, (iv) admission, (v) allocation of resources, (vi) occurrence of adverse event, (vii) death, (viii) patient exit (discharged patient or deceased patient), and (ix) resource deallocation.

In the proposed model, the queue is modeled by priorities. The moment resources are available, the priority patient is evaluated by the intensivist. The patient’s hospitalization admission or refusal depends on prognosis and potential benefit generated by therapeutic interventions. The admission criteria and discharge from the intensive care unit are established according to the ICU Admission, Discharge, and Triage Guidelines, accepted by the Society of Critical Care Medicine^52^. Thus, patients referred for ICU admission are prioritized according to their condition, as expressed in Table 1.

**Table 1:** Patient admission priority classification.

Admission priority	Recovery probability	Therapeutic support limitation	Need for intervention	Need for monitoring
1	High	No	Yes	Yes
2	High	No	No	Yes
3	Low	Yes	Yes	Yes
4	Low	Yes	No	Yes
5	Null	Yes	Yes	Yes

Patients classified as priority 2 and priority 4 according to Table 1 have a high risk of needing immediate intervention, so they should be monitored. Patients assessed as priority 5 are usually terminally ill, dying or potential organ donors, indicated to be in ICU only when there is a medical specification. The intensive care unit is an open system in which patients can arrive at any time. Thus, the number of patients in the queue and resource usage vary over time. Here, we propose ICU performance metric such as the relationship between the number of admissions $n_a$, length of stay $l_s$ in days, number of patients in queue $n_q$, and number of beds in use $n_b$. These variables are measured as average for the whole unit during a given period of time. The ICU performance $\eta _{icu}$ expression is given by:

5 $$\begin{aligned} \eta _{icu} = \dfrac{\dfrac{n_a}{n_{a_{ref}}} + \dfrac{l_{s_{ref}}-l_s}{l_{s_{ref}}} + \dfrac{n_{q_{ref}}-n_q}{n_{q_{ref}}} + \dfrac{n_b}{n_{b_{ref}}}}{4}, \end{aligned}$$

where the variables $n_{a_{ref}}$, $l_{s_{ref}}$, $n_{q_{ref}}$ and $n_{b_{ref}}$ are fixed at the maximum reference value of variables $n_a$, $l_s$, $n_q$ e $n_b$, respectively. The variable $n_{a_{ref}}$ is calculated following the steps: (i) to calculate the length of stay distribution that represents all patients based on the frequency and the length of stay related to the patients priority, (ii) to weigh the adverse events cases considering the extension of stay in the ICU, (iii) to calculate the average length of stay in ICU, (iv) to divide 365 (days of a normal year) by the average length of stay in ICU and (v) to multiply by the number of ICU beds. The variable $l_{s_{ref}}$ is determined by applying the steps: (i) to get the length of stay distribution representing all patients, (ii) to define the number of standard deviations that will be added to the mean of the distribution, (iii) to weigh the adverse events cases considering the extension of stay in the ICU. The variable $n_{q_{ref}}$ is calculated following the steps: (i) to define critical arrival rates distributions, (ii) to calculate the average arrival rate, (iii) to calculate the minimum number of patients treated in the ICU during one year considering the maximum reference length of stay $l_{s_{ref}}$, (iv) to calculate $n_{q_{ref}}$ as the difference between the total demand for ICU beds and the minimum number of patients treated in the ICU. We recommend that the variable $n_{b_{ref}}$ be defined based on the medical literature.

The ICU parameters sensitivity are: number of beds $n_{bed}$, percentage of equipment $n_{equipment}$, percentage of staff $n_{staff}$, adverse event rate $r_{ae}$ and arrival rate mean $\mu _{arrival}$. Using the ICU model illustrated in Fig. 4, the base scenario or base case $\beta$ is defined as the optimized system configuration case: maximum number of resources $n_{bed}$, $n_{equipment}$ and $n_{staff}$ available in the ICU to meet demand given by $\mu _{arrival}$ (obtained empirically) and adverse event rate equal to the sum of preventable event rate and non-preventable event rate, in which the preventable event rate is *zero* and the non-preventable event rate is given by the medical literature. The complexity measure is calculated using (4) during the simulation of the model illustrated in Fig. 4. Each patient in the queue adds a connection to the system and when hospitalized the patient adds three to four connections. After admission, the patient connects with the bed, the equipment, the staff, and the adverse events (if affected by any adverse event).

## Results

The ICU base scenario for simulation consisted of 10 beds, $100\%$ of equipment, and $100\%$ of the staff workload, with a $5\%$ addition margin. This margin refers to cases in which the professional needs to extend their working hours, for example, cardiac resuscitation close to the staff’s shift change. Considering the knowledge of specialists, patients were ranked by priority, as follows: $35\%$ for Priority 1, $50\%$ for Priority 2, $7\%$ for Priority 3, $7\%$ for Priority 4, and $1\%$ for Priority 5. The waiting queue type was first in, first out (FIFO), conditioned to the priority order. The admission refusal rate was set at $10\%$. For each admitted patient, the following were allocated: one bed, from $6\%$ to $12\%$ of the total equipment and from $6\%$ to $12\%$ of the staff’s workload, following a uniform distribution *U*(6, 12) for equipment and staff. The ICU average length of stay, in days, was related to the patients priority^53^, represented by a normal distribution equal to: *N*(8, 3) for Priority 1, *N*(5, 2) for Priority 2, *N*(7, 1) for Priority 3, *N*(7, 1) for Priority 4 and *N*(30, 7) for Priority 5.

Based on the medical literature^13–15^, the adverse event rate was set at $12\%$. For each adverse event occurrence, the patient length of stay was increased by a period between 15 and 45 days, represented by a *U*(15, 45) uniform distribution. The ICU death rate was set at $20\%$. The patient leaves when he or she is discharged or dies, situations in which it’s reserved resources are deallocated. For cases of admission refusal, the patient leaves the system before entering the ICU room. Patients who received intensive care can be classified as (i) recovered with no adverse event history, (ii) deceased with no adverse event history, (iii) recovered with adverse event history, and (iv) deceased with adverse event history. In the base scenario or base case, the patient arrival rate was represented by a normal distribution with 36 hours mean and 4 hours standard deviation, *N*(36, 4). In other words, every 36 hours on average there is one ICU bed request. All simulations were performed using the Simulink computational tool from MATLAB$^{\copyright }$ software.

The system performance was measured using the expression (5). We calculated the variables $n_{a_{ref}}$, $l_{s_{ref}}$, $n_{q_{ref}}$ and $n_{b_{ref}}$ through experiments considering the aforementioned distributions. The maximum reference number of admissions $n_{a_{ref}}$ was calculated based on the mean of the length of stay distribution *N*(6.58, 2.26), which represents all patients. Considering the adverse event rate, $88\%$ of the total number of patients admitted stay in ICU for an average of 6.58 days, and $12\%$ stay for an average of 36.58 days since the ICU length of stay in these cases increases an average of 30 days. Therefore, the average length of stay is 10.18 days. In one year, 360 patients, approximately, are admitted into an ICU with 10 beds.

The value $l_{s_{ref}}$ was equal 17.6 days, as a result of the steps: (i) to get the length of stay distribution representing all patients, (ii) to add three standard deviations to the mean value of the length of stay distribution, (iii) to weight the adverse events cases. The obtained values from the steps were: (i) *N*(6.58, 2.26); (ii) 14 days; (iii) 17.6 days. We added three standard deviations ($3\times 2.26$) to the mean value (6.58) resulting in approximately 14 days. As $12\%$ of patients have adverse events, the maximum reference length of stay $l_{s_{ref}}$ resulted in 17.6 days, considering that $88\%$ of total patients would be hospitalized for 14 days and $12\%$ of patients would stay in the ICU for about 44 days.

Then, the maximum reference queue size $n_{q_{ref}}$ was calculated, considering critical arrival rates: *N*(4, 4), *N*(6, 4) and *N*(12, 4). The average of these arrival rates means resulted in 7.33 hours, which would correspond to an average of 3 patients arriving per day and 1194 patients per year (simulated period) approximately. If a minimum number of patients were treated during the year, that is, when each patient length of stay was maximum as reference (17.6 days), the total number of patients who would go into an ICU with 10 beds would be 207. Thus, the maximum reference queue size would be 990 patients approximately.

We define the reference for the maximum reference number of beds $n_{b_{ref}}$ in use as $85\%$ of the total beds. Thus, the bed availability is kept within the expected limits for the ICU, according to McManus^16^. Since the modeled ICU has 10 beds, whenever the mean number of beds in use exceeds 8.5, the expression (6) determines the fourth installment numerator of performance metric. This modeling is a way to penalize situations in which the ICU is working at its limit.

6 $$\begin{aligned} \left\{ \begin{array}{lll} n_b &{} \textit{if} &{} n_b < 8.5\\ n_{b_{ref}} - 5 \cdot (n_b-n_{b_{ref}}) &{} \textit{if} &{} n_b \ge 8.5 \\ \end{array} \right. \end{aligned}$$

Table 2 summarizes the calculated values for the variables $n_{a_{ref}}$, $l_{s_{ref}}$, $n_{q_{ref}}$ and $n_{b_{ref}}$, which represent maximum reference values for the variables $n_a$, $l_s$, $n_q$ and $n_b$, respectively.

**Table 2:** Reference values for variables $n_a$, $l_s$, $n_q$ and $n_b$.

Variable	Value	Description (maximum mean value of...)
$n_{a_{ref}}$	360	...Admissions number
$l_{s_{ref}}$	17.6	...Length of stay
$n_{q_{ref}}$	990	...Number of patients in queue
$n_{b_{ref}}$	8.5	...Number of beds in use

After defining probabilistic distributions and reference values for calculating system performance, we performed the ICU parameters sensitivity study: $n_{bed}$, $n_{equipment}$, $n_{staff}$, $r_{ae}$ and $\mu _{arrival}$, relative to the normal distribution with standard deviation equal to 4 hours.

### Case study 1: ICU model sensitivity analysis

The ICU model sensitivity analysis was developed using the area method. The base value for number of beds $n_{bed} = 10$, percentage of equipment $n_{equipment} = 100\%$, percentage of staff $n_{staff}=105\%$, adverse event rate $r_{ae}=12\%$ and arrival rate mean $\mu _{arrival}=36h$. These values were defined empirically, considering the desired operating conditions of the ICU (optimized base case). The scenarios are built modifying the parameters $n_{bed}$ (from 1 to 20 beds), $n_{equipment}$ (from $15$ to $200\%$), $n_{staff}$ (from $15$ to $200\%$), $r_{ae}$ (from $12$ to $24\%$) and $\mu _{arrival}$ (from 4 to 72*h*).

We simulate the ICU operation for a one-year period. Considering the ICU optimized configuration (base case), an average of 193.79 patients were admitted to the ICU, who stayed hospitalized for about 8.49 days. The queue mean size was 5.69 patients with approximately 8.67 beds utilization during the simulated period. Thus, the value of the variables was equal to $n_a = 193.79$, $l_s = 8.49$, $n_q = 5.69$ and $n_b = 8.67$. The performance mean value calculated using the expression (5) was $74\%$.

**Figure 6:**
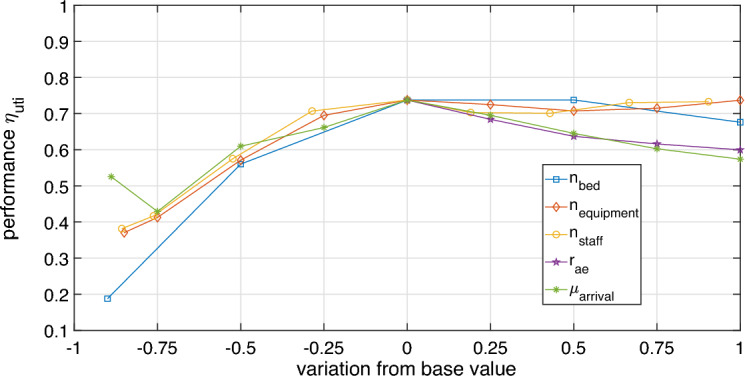
Spider-diagram of the ICU system for optimized base case.

Figure 6 refers to the base case spider diagram, in which the ICU working condition is regular. In Fig. 6, the number of beds $n_{bed}$ negative variation (in blue) generated the greater impact on performance. The parameters percentage of equipment $n_{equipment}$ (in orange) and staff percentage $n_{staff}$ (in yellow) showed similar behavior, with a significant impact for values below the base value. The adverse event rate $r_{ae}$ (in purple) was only varied from $0\%$ to $100\%$, since the estimated base value refers to non-preventable events. The base value $r_{ae}$ was equal to the smallest feasible value for the model in question. The increase in adverse event rate by $100\%$ led to a drop in performance greater than $10\%$. The arrival rate parameter $\mu _{arrival}$ mean variation (in green) led to lower performance values in both contexts analysis (negative variation and positive variation).

From one-at-a-time measures, visually presented in the spider diagram in Fig. 6, the area method was applied to calculate the sensitivity indices. The values obtained were: (i) $S_{n_{bed}}^a = 0.2556$, (ii) $S_{n_{equipment}}^a = 0.1904$, (iii) $S_{n_{staff}}^a = 0.1757$, (iv) $S_{r_{ae}}^a = 0.1121$, and (v) $S_{\mu _{arrival}}^a = 0.2662$. The most sensitive parameter was $\mu _{arrival}$, followed by the $n_{bed}$. The sensitivity index values are used to define system connections relevance values. In this way, after completing the sensitivity analysis step, we can calculate the ICU system complexity.

### Case study 2: ICU system complexity calculation

The complexity metric application using the expression (4) depends on the probability *P*(*c*) definition and the relevance $\gamma _c$ of the system connections. The probabilities of each connection type in the ICU system are presented in Table 3. The queue connection probabilities were defined experimentally considering each patient priority. The experimental probability refers to the number of patients per priority in the queue at each analyzed instant in regard to the total number of patients in the queue. Probability values for resources and adverse events were defined according to the base case configuration.

**Table 3:** Occurrence probabilities of connections in ICU system.

Connection *c*	*P* (*c*)
Patient priority 1 – queue	0.06
Patient priority 2 – queue	0.40
Patient priority 3 – queue	0.19
Patient priority 4 – queue	0.29
Patient priority 5 – queue	0.06
Patient – bed	0.10
Patient – equipment	0.09
Patient – staff	0.09
Patient – adverse event	0.12

Considering the ICU configuration with 10 beds, the patient connection probability to the bed is 0.1. Thus, each patient requires an average of 0.09 of the total equipment and the total team workload. This value 0.09 refers to the uniform distribution *U*(6, 12) mathematical expectation, used to define the equipment or staff quantity required by each patient. As for adverse events, the connection probability refers to its rate, which is 0.12.

The system connections relevance $\gamma _c$ was defined based on the parameters sensitivity indices. We check which parameters directly influence each connection and thus define the relevance value $\gamma _c$, as shown in Table 4.

**Table 4:** Connections relevance of the ICU system.

Connection *c*	$\gamma _c$
Patient – queue	$S_{\mu _{arrival}}^a$
Patient – bed	$S_{n_{bed}}^a$
Patient – equipment	$S_{n_{equipment}}^a$
Patient – staff	$S_{n_{staff}}^a$
Patient – adverse event	$S_{r_{ae}}^a$

The base case complexity was $\psi ^{icu}(c,\gamma ) = 20.30$, considering the average obtained after 100 system simulation repetitions for a *one* year period. The obtained complexity value can be understood when compared to complexity values from other scenarios. We modified the arrival rate mean $\mu _{arrival}$ to analyze overload cases, as shown in Table 5. The impact of changing the arrival rate was verified by simulating the scenarios $\beta ^{12h}$ and $\beta ^{24h}$, since the number of resources and adverse event rate were kept equal to the optimized base case $\beta ^{36h}$. The variables that make up the ICU performance measure, expressed by (5), were obtained after simulating each scenario and their values are shown in Table 6.

**Table 5:** ICU system overload and regularity cases.

Base case	Condition	$n_{bed}$	$n_{equipment}$	$n_{staff}$	$r_{ae}$	$\mu _{arrival}$
$\beta ^{12h}$	Overload	10	100%	105%	12%	12*h*
$\beta ^{24h}$	Overload	10	100%	105%	12%	24*h*
$\beta ^{36h}$	Regularity	10	100%	105%	12%	36*h*

**Table 6:** Simulation data of ICU performance components variables for overload and regularity cases.

Base case	Admissions $n_a$	Length of stay $l_s$[days]	Patients in queue $n_q$	Beds in use $n_b$	Performance $\eta _{icu}$
$\beta ^{12h}$	179.64	9.75	244.09	9.31	0.56
$\beta ^{24h}$	194.80	8.98	58.44	9.22	0.64
$\beta ^{36h}$	193.79	8.49	5.69	8.67	0.74

**Figure 7:**
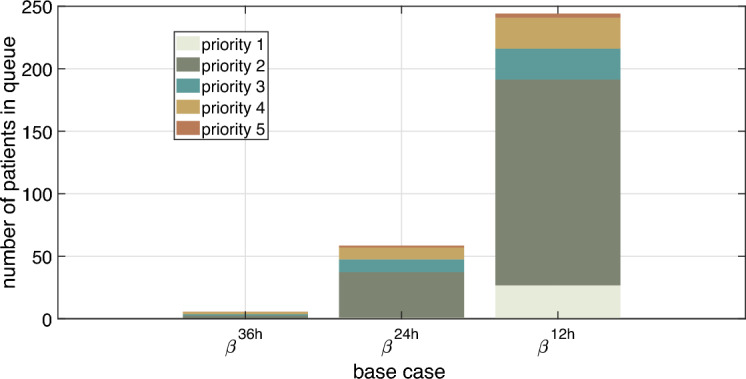
Number of patients per priority in the ICU queue for base cases $\beta ^{36h}$, $\beta ^{24h}$ and $\beta ^{12h}$.

Under overload condition, the performance values obtained were inferior to the optimized base case performance: $56\%$ relative to $\beta ^{12h}$ and $64\%$ relative to $\beta ^{24h}$. The Table 6 data indicate a queue size $n_q$ increase by more than $1000\%$ and ICU occupation above the recommended value ($85\%$), since the ICU had $93.1\%$ beds in use for the case $\beta ^{12h}$ and $92.2\%$ for the case $\beta ^{24h}$. The average number of patients in the queue according to each priority is shown in Fig. 7 and in Table 7. As the available resources were insufficient to meet the demand, the number of patients in the queue increased significantly (and in a non-linear manner) with the arrival rate mean reduction.

**Table 7:** Number of patients by priority in ICU queue relative to overload and regularity cases.

Patient priority	Number of patients in queue		
	$\mathbf {\beta ^{12h}}$	$\mathbf {\beta ^{24h}}$	$\mathbf {\beta ^{36h}}$
1	26.71	0.97	0.38
2	164.72	36.30	2.16
3	24.62	10.28	1.07
4	24.66	9.44	1.80
5	3.36	1.42	0.25

Based on Fig. 7 and Table 7, we verified that the largest number of patients in the queue refers to priority 2 patients. However, regarding the regularity case, these patients represented $38.01\%$ of the total patients in the queue, while in overload cases, priority 2 patients represented more than $60\%$ of the total patients in the queue. The lowest values in Table 7 are priority 1 and priority 5 patients. Most of the priority 1 patients occupied ICU beds while priority 5 patients were the smallest group with ICU beds demand. Both priority 3 and priority 4 patients percentages increased as the arrival rate mean also increased.

The calculated complexity values for the scenarios $\beta ^{12h}$, $\beta ^{24h}$ and $\beta ^{36h}$ are shown in Table 8. The complexity of these scenarios was 3 to $7.5\times$ greater than the regularity scenario $\beta ^{36h}$ complexity. The increase in overload cases complexity is due to the patient increase in the system. These patients may be waiting for bed in the admission queue due to resource unavailability or inside the unit, generating a high resource utilization rate.

**Table 8:** Complexity values to analyzed scenarios.

Scenario	$\psi ^{icu}(c,\gamma )$
$\beta ^{12h}$	153.42
$\beta ^{24h}$	62.34
$\beta ^{36h}$	20.30

## Discussion

In this work, we built a computational model of an intensive care unit and simulate it. The developed model considered internal elements of the system and external aspects, described as input parameters and output variables. Initially, we verified how sensitive the system was to the variation of the input parameters. Thus, we could see that the sensitivity was distributed among all parameters. The calculated sensitivity indices presented values between $11$ and $27\%$, that is, the least sensitive parameter is relevant to the system as well as the most sensitive parameter. In other systems, it is common to verify sensitivity indices of certain parameters close to *zero* and others, above $50\%$^48,51,54,55^.

The sensitivity indices were used in the proposed complexity measure to weight the connections between the system elements. The use of the local sensitivity analysis method in the methodology corresponds to a limitation in the metric, especially if the base case is in a instability region. To resolve this limitation, the application of the global sensitivity analysis method is recommended as an alternative to the area method^56,57^. However, we emphasize that if the system is in a stability region, the local sensitivity analysis is indicated, especially when the complexity metric is applied to a real system model, dispensing with the computational model.

Considering the ICU system relevance for health recovery, the system as a whole must show reliability and robustness. The ICU system shows robust behavior when: (i) the adverse event occurrence is reduced, (ii) the patients flow occurs regularly, and (iii) the proper functioning is maintained even in instability situations, such as unexpected demand increase, lack of human resources or equipment failure. To promote ICU system robustness, actions can be adopted, such as functional overlap between health professionals or the use of multifunctional critical care apparatus^58–60^.

We can investigate aspects related to ICU system robustness by observing the complexity variation. System changes, such as idleness or overload, can affect the parameters sensitivity and the connections entropy. The robustness analysis associated with the specialists expertise in the critical care area contributes to decision-making in ICU systems management. These decisions can improve resource allocation and adjust the system’s operating rules.

The proposed complexity metric has a potential impact on the practice of intensive care in terms of resource management based on demand. The decision-making process based on the complexity of the system can be performed by hospital managers or even managers of a nation, in case the government wants to know the possibilities of acting in emergencies, such as a pandemic. The relevance of using this complexity score lies in its ability to integrate different system characteristics such as configuration, arrangement, performance, and workload. Monitoring the parameters of the ICU system to apply the proposed complexity metric can be a way to avoid deaths and financial losses in the context of intensive care.

Using simulation, the ICU system exposure to high demand regimes was important to observe the considerable increase in complexity $\psi ^{icu}(c,\gamma )$ and loss of system efficiency checked through the variables number of admissions $n_a$, length of stay $l_s$, patients in queue $n_q$, beds in use $n_b$ and performance $\eta _{icu}$. Overload situations in ICU systems can occur both during their routine operation and in exceptional situations, such as the current COVID-19 pandemic. Individuals contaminated with new coronavirus or SARS-CoV-2 can present symptoms with varying degrees of severity, from asymptomatic to serious cases, in which the patient develops acute respiratory syndrome accompanied or not by other vital systems impairment. In severe cases, the patient’s admission to the ICU is recommended^61,62^.

The high number of serious COVID-19 cases recorded in several countries in recent years has caused a situation of generalized overload in health systems at a global level, especially those aimed at intensive care^63^. ICU occupation rates above $85\%$ – reaching $100\%$ in numerous cases – have been observed since the pandemic beginning^64^. The ICU systems overload is addressed in studies performed in hospitals in China^65^ and the United States, the main pandemic epicenters^66,67^, in Italy, one of the most affected countries by the pandemic in contaminated percentage^68,69^, in the Netherlands, Germany^69^ and around 216 countries analyzed by Rocks and Idriss^70^ and Sen-Crowe et al.^63^.

From the hierarchical organization of complex systems point of view, the ICU system has subsystems meanwhile is part of a larger system: the hospital system. The ICU complexity increase can affect the entire hospital system complexity. The proposed complexity metric can be used to evaluate the subsystem complexity relationship with the system that comprises it. Regarding the internal dynamics, the proposed metric can contribute to the complexity evaluation when there are system changes, such as lack of beds, lack or failure of equipment, and lack of professionals.

## Conclusion

This work proposed a system complexity metric based on weighted connections. We use sensitivity indices to weight the connections between system components. Thus, the influence of the external environment could be verified through sensitivity analysis. The simulation of the intensive care unit system provided us: (i) average number of patients admitted, (ii) average ICU length of stay, (iii) number of patients by priority in the queue, and (iv) system resource usage. Using the simulation data, we calculate the system performance, and the ICU systems parameters sensitivity and complexity, under regularity and overload conditions. The intensive care unit presents complex system characteristics, as the interaction between its components generates functionality (critical care) that is impossible to obtain by its parts separately. In addition, the ICU system has the self-organizing characteristic, observed when professionals need to extend their working hours or when there is a need for overlapping functional competencies. The hierarchical organization is also observable, given that the system’s functioning and structures can be analyzed at different levels. Based on the results obtained in this work, we list some future investigations: (i) sensitivity analysis and complexity study of idleness cases and system overload in comparison with the regularity case, (ii) analysis regarding performance measure relations and system complexity, and (iii) metrics development to define regions of system robustness.

## Data Availability

Some or all data, models, or code that support the findings of this study are available from the corresponding author upon request.
